# Perceived Discrimination and Motivation to Pursue Higher Education in Ethiopian-Origin Students: The Moderating Role of Ethnic Identity

**DOI:** 10.3389/fpsyg.2021.647180

**Published:** 2021-06-08

**Authors:** Ortal Slobodin, Tamar Icekson, Lee Herman, Ofri Vaknin

**Affiliations:** ^1^Department of Education, Ben-Gurion University, Beer-Sheva, Israel; ^2^Department of Management, Ben-Gurion University, Beer-Sheva, Israel; ^3^School of Behavioral Sciences, Peres Academic Center, Rehovot, Israel

**Keywords:** academic motivation, Ethiopian-origin students, ethnic identity, high education, perceived discrimination

## Abstract

Research has increasingly recognized the adverse effects of perceived discrimination on the academic outcomes of children and adolescents from ethnic and racial minority backgrounds. However, little is known about the association between perceived discrimination and the motivation of ethnic minority students to pursue higher education. Guided by an academic resilience framework, the current study examined the relationship between perceived discrimination and two types of motivation to pursue higher education (personal/career-driven and expectation-driven) among Ethiopian undergraduate students in Israel. In addition, we examined the role of ethnic identity as a potential moderator of this relationship. Participants were 183 undergraduate students of Ethiopian origin (77% females) who studied in 18 different higher-education institutes. Participants completed self-report questionnaires concerning their experiences of perceived discrimination, affiliation with their Ethiopian identity, and their reasons for pursuing higher education. Results showed that frequent discrimination experiences were positively related to a stronger ethnic identity and to higher levels of personal/career motivation to pursue higher education. Ethnic identity moderated the relationship between perceived discrimination and personal/career motivation so that the association was significant under low and moderate levels of ethnic identity but not under high levels. Our findings suggest that the awareness of discrimination may motivate students to pursue higher education and succeed in academia. However, the motivating force of perceived discrimination diminishes under high levels of ethnic identification. Further investigation is needed to explore how discrimination and ethnic identity work together to impact academic motivation in different developmental stages and socio-cultural contexts.

## Introduction

Ethnic and racial differences in higher education enrollment and performance have been extensively described in the literature (Stevenson and Whelan, 2013; Schnell and Azzolini, 2015; Frings et al., 2020). Despite the increasing numbers of racial and ethnic minority students in the higher education system and their growing academic success (Fletcher and Tienda, 2010; Hotchkins and Dancy, 2015), these students still have lower academic achievements and higher dropout rates than their non-minority peers (Cao and Maloney, 2018; for review, see Woolf et al., 2011). These racial and ethnic gaps were previously attributed to multiple barriers, including inadequate high-school preparation, language challenges, and financial difficulties (Stegers-Jager et al., 2016; Obinna and Ohanian, 2018). For decades, researchers have explored the effects of discrimination and social marginalization as a possible explanation for the ethnic and racial gaps in academic outcomes (Berwise and Mena, 2020).

Perceived discrimination may be experienced as a chronic, traumatic, interpersonal stressor (Polanco-Roman et al., 2016), particularly during emerging adulthood (Kessler et al., 1999; Pérez et al., 2008). Exposure to discriminatory behaviors may deplete social and personal resources and have a negative effect on mental and physical health (Paradies et al., 2015; Banerjee et al., 2018) despite the growing literature on the influence of discrimination on educational outcomes, it is not clear how perceived discrimination is associated with minority students’ motivation to pursue higher education and what are the underlying mechanisms of this link.

The academic resilience perspective (Arellano and Padilla, 1996) provides an appealing framework for understanding the factors that promote or impede academic success among individuals who face adversity. This perspectives views perceived discrimination as a significant risk factor that can negatively affect academic outcomes. The academic resilience framework will be used here to examine how perceived discrimination is associated with different types of academic motivation among Ethiopian undergraduate students. In addition, we considered whether and how students’ levels of ethnic identification moderate the effects of perceived discrimination on their motivation to pursue higher education.

### Ethnic Minority Students’ Motivation to Pursue Higher Education

Broadly, motivation refers to a process whereby goal-directed behaviors are initiated and maintained (Pintrich and Schunk, 2002). Research on academic motivation has predominantly focused on the reasons for students’ engagement in specific tasks, and less attention has been devoted to the students’ long-term goals, such as acquiring higher education or obtaining an academic degree (Teowkul et al., 2008; Knutsen, 2011). Cote and Levine (1997) distinguished five types of goals that motivate students to attend college: career goals, including obtaining a good job, success, and financial rewards; personal goals, such as developing oneself personally or intellectually; humanitarian goals, such as helping others or improving the world; expectation driven goals, such as meeting the expectations of others; and default motivation (avoiding other less desirable options). They found that reasons based on career goals or personal development in the first year of college positively predicted self-management, self- motivation and academic performance 2 or 3 years later. In contrast, attending college for lack of other alternatives (i.e., by default) negatively predicted self- motivation and academic performance (Cote and Levine, 1997).

Investigating ethnic minority students’ reasons for acquiring higher education is especially crucial because such reasons influence academic engagement and eventually determine which students persist with their studies (Isik et al., 2017; Milovanska-Farrington, 2020) however, research focusing on the academic motivation of minority students in higher education is severely limited and currently hindered by the lack of data on non-United States population. In a recent literature review of academic motivation in ethnic minority students (Isik et al., 2018), only eight of the 45 included studies were conducted in the context of higher education. Of these eight studies, seven were conducted in the United States and one in New Zealand (Gavala and Flett, 2005). The few existing studies have shown that minority students’ reasons for acquiring higher education are influenced by their cultural values, such as family interdependence and ethnic identification (Phinney et al., 2006). Moreover, these studies suggested that minority students’ reasons to pursue higher education are associated with both individual concerns (e.g., intellectual curiosity, personal interest, a desire to obtain a satisfying and rewarding career) and collective concerns (meet the expectations of the family, preserve connections with their family, community and culture) (Dennis et al., 2005; Phinney et al., 2006; Knutsen, 2011) in a large sample of ethnically diverse students (Latino, Asian American, African American, and European American), Phinney et al. (2006) found that both career/personal motivation and humanitarian motivation were related to three college adjustment variables: college self-efficacy, self-confidence in reaching one’s goals, and commitment to the college. Default motivation, on the other hand, had relatively low levels of importance. Importantly, Phinney et al. (2006) noted that career and personal intellectual goals tend to converge for minority students because, for students of low and medium socioeconomic status (SES), personal development is often linked to a desire for social mobility. In the same vein, Dennis et al. (2005) found that both personal/career and family expectation motivations were highly endorsed by minority college students. However, only the personal/career motivation was related to college adjustment and commitment.

Family expectations may have positive and negative effects on the higher-education adjustment of ethnic minority students. In a qualitative study by Hernandez (2000), Latino students associated their academic persistence with family’s encouragement and fear of parental disappointment. These students were motivated to graduate from college because they “owed a debt” to their parents, who often worked hard to allow them to acquire higher education (Hernandez, 2000). However, family and peer expectations may also have adverse effects on students’ motivation if they conflict with academic values and obligations. Saenz and Ponjuan (2009) noted that in families of Latin origin, the tight solidarity may lead individuals to sacrifice their own needs for those of the family. Males, especially, are expected to protect their families and help support them. Therefore, higher education is not part of their future development.

A recent study of Hmong American high-school students of low-income backgrounds, Lor (2019) described how strong family connectedness binds both male and female students to gender-based expectations and obligations that prevent them from pursuing opportunities for social mobility. This, and the above-mentioned studies of Latino students emphasize the need to explore the relationship between socio-cultural and contextual factors and minority students’ motivation to pursue higher education (Gizdarska, 2017).

### Perceived Discrimination and Academic Motivation

Consistent with the academic resilience perspective (Arellano and Padilla, 1996), prior studies have found that perceived discrimination was negatively associated with academic motivation in adolescents and emerging adults (Wong et al., 2003; Eccles et al., 2006; Alfaro et al., 2009). For instance, Wong et al. (2003) showed that African American adolescents’ experiences of racial discrimination at school were associated with decreased levels of two motivational dimensions: one’s perception of the usefulness of a particular task for future goals and one’s evaluation of competence in a particular area. Alfaro et al. (2009) found that experiences of discrimination were negatively associated with academic motivation, both concurrently and longitudinally, for Latino male adolescents, but not females. Likewise, Byrd (2015) found negative associations between peer and teacher discrimination and intrinsic academic motivation (internal drive or pleasure that motivates behaviors or accomplishments.) in African American youth. These findings support Neblett et al.’s (2006) view that discrimination might lead to heightened feelings of vulnerability, which, in turn, lead to a decrease in intellectual curiosity, academic self-esteem, and academic persistence. However, a recent systematic review found that discrimination may also be associated with increased academic motivation (Isik et al., 2018), probably because it drives ethnic minority students to overcome adversity and exceed in their education (Perreira et al., 2010). For example, Reynolds et al. (2010) found that institutional racism-related stress was positively correlated with intrinsic academic motivation and negatively correlated with extrinsic motivation (behave for some external rewards or outcomes) among Latino and African American college students.

To date, research on the relationship between perceived discrimination and academic motivation has largely focused on children and adolescents, and little is currently known about the correlates of perceived discrimination in later stages of the academic venue when young adults decide to pursue higher education. Moreover, it is not clear whether and how perceived discrimination is associated with different types of motivation to pursue higher education. Previous research linked perceived discrimination in school with a reduction of students’ academic orientation, self- efficacy, and curiosity (O’Hara et al., 2012; Banerjee et al., 2018; Leath et al., 2019) as well as with a reduction of student’s career self-efficacy and vocational outcome expectation (Conkel-Ziebell et al., 2019). Following this line of research, we argue that young adults who were exposed to discriminatory experiences throughout their school years would have lower academic and career self-expectations and consequently, reduced levels of personal/career motivation to pursue higher education.

In addition to its negative association with academic and career self-attitudes and beliefs, perceived discrimination, with its detrimental effect on family relationships, may be linked to reduced levels of expectation-driven motivation to pursue higher education (Kwon, 2020). Perceived discrimination has been associated with poorer parent-child relationships and with increased family conflict (Riina and McHale, 2010; Bécares et al., 2015). More specifically, perceived discrimination was associated with lower parental expectations of their children. In a study of African American mothers of adolescents (Varner and Mandara, 2013), mothers with more discrimination concerns were less responsive and monitoring and reported more conflict with their adolescents. Because these mothers also had lower academic expectations for their sons, they socialized their children in a manner that was less focused on the academic path. Following these findings, we suggest that the exposure to discriminatory experiences would negatively affect expectation-driven motivation to pursue higher education not only due to its adverse effects on family ties, loyalties, and obligations but also because children may internalize their parents’ low expectations.

### The Moderating Role of Ethnic Identity

According to the academic resilience perspective, resilience factors have various effects on the relationship between risk factors and academic adjustment, one of which is by serving as moderators between risk factors and outcomes (Alfaro et al., 2009; García-Izquierdo et al., 2018). Researchers have examined ethnic identity as an individual difference variable that may modify the strength or the direction of discrimination (Lee et al., 2015).

Ethnic identity has been formulated differently by psychologists, sociologists, and anthropologists. In the current study, we use a developmental psychology perspective (Phinney, 2003), where *ethnic identity* is defined as “a feeling of belonging to one’s group, a clear understanding of the meaning of one’s membership, positive attitudes toward the group, familiarity with its history and culture, and involvement in its practices” (Phinney et al., 1994)^1^.

Ethnic identity conceptualizations have theoretical underpinnings in social identity theory (Tajfel and Turner, 1986), positing that members of low-status social groups may maintain psychological well-being in the face of discrimination by becoming more highly identified with their socially devalued in-group (Tajfel and Turner, 1986). According to this view, the negative impact of perceived discrimination on psychological health may be buffered for those people who highly identify with their ethnic group (Phinney, 2003). In line with Phinney’s (2003) ethnic identity moderation hypothesis, the current literature consistently emphasizes the protective role that positive perceptions of ethnic identity play in the mental health and adjustment of ethnic and racial minority youth (Umaña-Taylor and Updegraff, 2007; Torres and Ong, 2010; Galliher et al., 2011). In the context of academic motivation, research in African American adolescents have associated a high level of African self-consciousness to a higher level of achievement motivation and feelings of responsibility for the African American community (Robinson and Biran, 2006; Wang and Eccles, 2013). For instance, Byrd and Chavous (2011) found that a positive racial school climate and positive messages about race were associated with higher rates of achievement motivation in 11th-grade African American students. However, research also suggests that high ethnic identification may hinder academic motivation due to increased awareness of negative stereotypes, lack of role models, and little emphasis on positive societal depictions of the ethnic group (Oyserman, 2008). Accumulated experiences of discrimination may force ethnic minorities to develop an oppositional identity, which rejects the values of the dominant culture (The rejection-identification hypothesis; Branscombe et al., 1999). When minority students strongly identify with their socially devalued in-group, they may reject the effortful pursuit of academic excellence as “acting White” (Ogbu, 1988), perhaps to the point of withholding academic motivation and efforts (Wildhagen, 2011).

As indicated in previous studies, perceived discrimination is a multidimensional construct, that may take many forms and occur in many contexts. However, all forms of discrimination represent, to varying degrees, threats to specific social identity needs (e.g., belongingness, esteem, control), which relate to important motives for academic engagement and performance (Richman and Leary, 2009; Verkuyten et al., 2019). Therefore, in the current study we consider perceived discrimination as a threat to minority students’ motivation to pursue higher education, a threat that may be buffered by a positive ethnic identity.

### Students of Ethiopian Origin in the Israeli Higher-Education System

Immigrants from Ethiopia arrived in Israel in two waves of government-organized mass emigration, in 1984 and 1991. Since then, Ethiopian immigration to Israel has continued, and 818 new immigrants arrived in 2019. At the end of 2019, the Ethiopian population in Israel was 155,300 people, 87,500 immigrants, and 67,800 born in Israel to Ethiopian parents [Central Bureau of Statistics (CBS), 2019]. Most Ethiopian Jews in Israel belong to the Beta Israel community (“the house of Israel”), and the others to the Falash Mura, who converted from Christianity to Rabbinic Judaism upon their arrival to Israel. In Ethiopia, the Beta Israel community was isolated from mainstream Jewish communities and suffered religious persecution. During the 19th and 20th centuries, many community members were forced into Christianity and came to be known as the Falash Mura. Unlike the Beta Israel community, the Falash Mura do not meet the criteria for immediate and automatic Israeli Citizenship granted to Jews by the 1950 Law of Return due to uncertainties over their ancestral lineage according to Jewish law (The Knesset of Israel., 2005). Political controversy has left this issue unresolved for many years, until, in 2015, the government pledged to bring all members of the Falash Mura to Israel by the end of 2020. Following this decision, 2000 of the approximately 8000 Falash Mura members immigrated to Israel between December 2020 and March 2021 during the “Zur Israel” operation (The Ministry of Immigration and Integration, 2020).

Many of the Ethiopian immigrants came from a rural, often illiterate society, and the transition to modern, urban society was fraught with integration problems, including cultural gaps, poverty, and social isolation (Kurman and Ronen-Eilon, 2004). These problems were associated with a high incidence of mental illness, delinquency, depression, and suicide among first- and second-generation Ethiopian immigrants (Goldblatt and Rosenblum, 2007; Offer, 2007; Wilchek-Aviad, 2015). Within the educational context, it appears that high- school students of Ethiopian origin perform more poorly than other students in every academic category, including standardized tests, dropout rates, and matriculation (Central Bureau of Statistics (CBS), 2016). Furthermore, students of Ethiopian origin tend to approach higher education with lower achievements than their non-Ethiopian peers, both in terms of high-school matriculation scores and psychometric test scores (Kalnisky and Brenner, 2016; Fuchs and Friedman-Wilson, 2017). Unlike other immigrant and minority groups in Israel, for Ethiopians, these disparities are not entirely explained by lower SES (Feniger et al., 2021).

The number of Ethiopian-origin students in the Israeli higher-education system increased in the last two decades from 747 in 2000 to 3536 students in 2019/2020. In 2015/2016, Ethiopian youth composed 3.3% of the Jewish population aged 20–29 years, but only 1.2% of the higher- education Jewish student population. Moreover, the dropout ratio of Ethiopian students from universities is 12%, compared to 8% in the Jewish student population (Central Bureau of Statistics (CBS), 2016; Feniger et al., 2016). A recent report based on ten semi-structured interviews with Ethiopian students in Israeli institutes of higher education (Ben Simon et al., 2018) identified the importance of institutional, parental, and peer support as well as personal factors (optimism, persistence, motivation) in their academic engagement and success. Israeli Council for Higher Education (2017) acknowledged the need to make higher education more accessible to young adults of Ethiopian origin, and it set ambitious goals to enlarge the number of students enrolled in universities. Despite this ambitious plan, little is known about the personal and social factors that may hinder or promote academic motivation and performance among students of Ethiopian origin in the Israeli higher education system (Kalnisky and Brenner, 2016).

Previous qualitative and quantitative research within the Ethiopian Israeli community confirmed the role of both personal and group values in individuals’ motivation for integration and success (Horowitz and Mosher, 1997; Salamon et al., 2009; Walsh and Yonas, 2012; Fanta-Vagenshtein and Anteby-Yemini, 2016). Yet, to date, very few studies have directly addressed how these values are associated with the academic motivation of college and university students of Ethiopian origin.

Studies focusing on the correlates of ethnic identification among adolescents of Ethiopian origin found both negative (Walsh et al., 2012) and positive (Walsh et al., 2015) associations between strong ethnic identity and risk behaviors. Additionally, ethnic identity may interact with SES to affect mobility. Mizrachi and Zawdu (2012) found that working-class Ethiopian Israelis de-emphasize racial identity, whereas the middle class uses global black identity models. It has been suggested that upwardly mobile Ethiopian Israelis feel racist attitudes toward them most strongly (Amit, 2012), often because they continue to be seen as ‘Ethiopians’ by others (Walsh and Yonas, 2012). These mixed findings emphasize the need to examine the pathways through which ethnic identity is associated with academic motivation in this group.

### The Current Study

As indicated by the above findings, the association between perceived discrimination, ethnic identity, and the motivation to pursue higher education is just beginning to be explored and has not been clearly defined (Hackett, 2017). Prior research suggests that ethnic identity may be related to psychological well-being and thereby to higher aspirations and confidence in the ability to achieve personal goals. It may also act as a moderator between perceived discrimination and the motivation to pursue higher education because a positive connection to one’s ethnic group may protect minority students from the perceived experiences of discrimination (Phinney, 2003, Phinney et al., 2006). Therefore, considering students’ ethnic identity both as a predictor of the different reasons to pursue higher education and as a moderator of the link between perceived discrimination and the motivation to pursue higher education is important.

The aim of the current study was two-fold. First, we were interested in examining the relationship between perceived discrimination, ethnic identity, and two main types of motivation to pursue higher education (personal/career driven and expectation driven; Dennis et al., 2005) among undergraduate students of Ethiopian origin. Given the adverse effects of perceived discrimination on students’ psychological, physical, and educational outcomes (Banerjee et al., 2018; Cheng et al., 2020; Wong et al., 2020), we assumed that perceived discrimination would deplete both personal and family/community resources (Ghafoori et al., 2019), and thus be negatively associated with personal/career-driven and expectation-driven motivation.

We also expected that ethnic identity would be positively associated with reasons for pursuing higher education that reflect a sense of identity and self-esteem, such as personal/career motivation as well as with reasons that reflect strong family and community connectedness and obligation, such as expectation-driven motivation (Robinson and Biran, 2006; Young et al., 2011; Wang and Eccles, 2013).

The second aim of the study was to examine whether ethnic identity acts as a moderating mechanism in the association between perceived discrimination and the two types of academic motivation. Drawing on Phinney’s (2003) ethnic identity moderation hypothesis, we expected that greater ethnic identification, which motivates students to try harder and counteract their devaluation, would buffer the impact of discrimination experiences on academic motivation.

In line with previous research and theory, our research hypotheses were:

(1) Perceived discrimination will be negatively associated with personal/career-driven and expectation-driven academic motivation.
(2) Ethnic identity will be positively associated with personal/career-driven and expectation-driven academic motivation.
(3) Ethnic identity would moderate the relationship between perceived discrimination and the two types of academic motivation so that the relationship would be weaker when ethnic identity is stronger.

**FIGURE 1:**
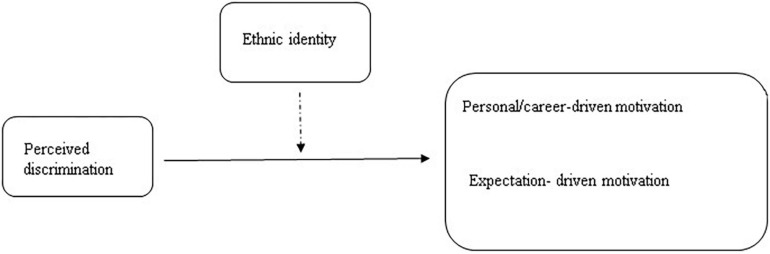
The theoretical model of the study. Ethnic identity as a moderator of the relationship between perceived discrimination and the two types of motivation to pursue higher education.

The conceptual model for this is presented in Figure 1.

## Materials and Methods

The study participants were 183 undergraduate students of Ethiopian origin, of whom 77% were women. Participants’ age was 20–45 years (*M* = 27.89, *SD* = 4.84). Most participants (73.2%) were single. Almost half of the participants (44.2%) were born in Ethiopia, and all others were native Israelis born to immigrant parents. Almost half of the participants were in their first academic year (42.6%), 18.6% were in the second academic year, 36.1% studied in their third year, and 2.7% in their fourth year or more. See Table 1 for demographic details.

**TABLE 1:** Participants’ background characteristics (*N* = 183).

		Mean	SD	*N*	%
Gender	Male			42	23
	Female			141	77
Marital status	Single			134	73.2
	Married			45	26.4
	Separated/divorced			2	1.1
	Widowed			1	0.5
	Other			1	0.5
Place of birth	Ethiopia			81	44.2
	Israel			102	55.8
Mother born in Ethiopia				148	80.9
Father born in Ethiopia				150	81.9
Academic year	First			78	42.6
	Second			34	18.6
	Third			66	36.1
	Fourth or more			5	2.7
Age		27.89	4.84		
SES^a^		2.56	0.86		

*^a^SES ranged from 1 (very low) to 5 (very high).*

To maximize the sample representativeness, we included students from 18 institutes of higher education in Israel – six universities, five academic colleges, five teacher education colleges, and two technological colleges. Participants were recruited through academic support units, digital communities of Ethiopian students (i.e., Facebook and WhatsApp groups), and contact persons in municipal, educational, and community programs for Ethiopian students. The study was approved by the Research Ethics Committee of Ben-Gurion University and was carried out according to the ethical standards of research with human subjects. Participation was voluntary, and all participants signed a written informed-consent form.

### Measures

All measures underwent a four-step cultural and language adaptation process (Weeks et al., 2007): (a) forward-translation from English to Hebrew by independent bilingual (English and Hebrew) individuals; (b) primary evaluation of the comprehensibility of the translated questionnaire; (c) back-translation into English; and (d) discussion and agreement on a final version. Additionally, all scales were culturally adapted by three research assistants of Ethiopian origin, and by a focus group of educators of Ethiopian origin.

*Background variables –* age, gender, and SES were recorded for each student. In addition, students’ high-school annual grade point average (GPA) or final matriculation scores were obtained.

*Perceived discrimination* was measured by the Experiences of Discrimination and Racism scale (Liebkind and Jasinskaja-Lahti, 2000). The scale examines the degree to which an immigrant has perceived discrimination or racism in a variety of settings. Using a four-point Likert scale, participants rated the degree to which they had encountered discrimination, difficulties, insult, or harassment because of their Ethiopian background in each of 10 settings (e.g., finding work, renting an apartment, in school, during military service). Inter-item reliability in the current study was α = 0.81.

*Ethnic identity* was measured by the acculturation scale (Shalom and Horenczyk, 2004) used previously with late Ethiopian adolescents in Israel (Walsh et al., 2012). The scale includes 11 items examining Ethiopian identity (e.g., “I feel connected to Amharic culture,” “I am pleased I come from Ethiopian heritage”). Items were rated on a five-point Likert scale. Inter-item reliability in the current study was α = 0.85.

*Motivation to acquire higher education* was assessed by using two subscales from the Student Motivations for Attending University (SMAU; Cote and Levine, 1997), which were found to be central to the study of minority students (Dennis et al., 2005): the personal/career-driven motivation and the expectation-driven motivation. Based on Phinney’s adaption of the scale for first-generation minority college students (Phinney et al., 2006), we combined the personal-intellectual reasons to pursue higher education with career-materialism reasons. The personal/career subscale (eight items) included items relating to personal- intellectual development and gaining more money, and finding a good job (inter-item reliability α = 0.79). Sample items in the personal/career scale include “Academic education would help me improve my intellectual capacity” and “Academic education would increase my chances of finding a good job.”

The expectation-driven subscale includes four items relating to pressure from family and others to pursue higher education (inter-item reliability α = 0.70). Sample items in the expectation-driven subscale include “There were pressures on me from my family to gain an academic degree” and “My parents will be disappointed if I didn’t succeed.” Participants were asked to rate, on a five-point scale (1 – *strongly disagree*, 5 – *strongly agree*), the degree to which they agree or disagree with the stated reason for acquiring higher education.

### Data Analysis

The three-stage analysis began with an analysis of descriptive statistics for all study variables. This step was followed by a correlational analysis which examined the relationships between discrimination, ethnic identity, and the two types of academic motivation. Third, to examine our main prediction regarding the moderating role of ethnic identity in the relationship between discrimination and academic motivation, we employed Hayes’s (2013) PROCESS macro (Hayes and Matthes, 2009). Background variables that were found to have significant associations with the main study’s variables (i.e., age, high-school GPA, and SES) were controlled. Levels of perceived discrimination, ethnic identity, and the two types of academic motivation were treated as independent variables, moderator, and outcomes, respectively. To have adequate power to detect a medium effect size in multiple regression, using a two-tailed test, with five predictors, α = 0.05, and power = 0.80 (Cohen, 1992), a minimum of 91 participants is required. Data analyses were carried out on SPSS Windows 25.0.

## Results

Results of the correlation matrix (Table 2) showed that, in contrast to the first hypothesis, perceived discrimination was positively associated with personal/career-driven motivation (*r* = 0.353, *p* < 0.001) but not with expectation-driven motivation (*r* = 0.095, *p* = 0.358). In line with the second hypothesis, ethnic identity was positively associated with both personal/career-driven motivation (*r* = 0.217, *p* = 0.016), and expectation-driven motivation (*r* = 0.274, *p* = 0.002). Discrimination was positively associated with ethnic identity (*r* = 0.423, *p* < 0.001).

**TABLE 2:** Pearson correlations between main research variables (*N* = 183).

Variables	1	2	3	4	5	6	7
1. Age	1	−0.137	−0.324***	−0.049	0.139	0.016	−0.205*
2. SES		1	0.032	0.040	−0.216**	−0.005	0.146
3. High school GPA			1	0.063	−0.090	0.085	0.044
4. Perceived discrimination				1	0.423***	0.353***	0.095
5. Ethnic identity					1	0.217*	0.274**
6. Personal/career-driven motivation						1	0.047
7. Expectation-driven motivation							1

*p < 0.05. **p < 0.01. ***p < 0.001. SES, socioeconomic status; GPA, grade point average.

**FIGURE 2:**
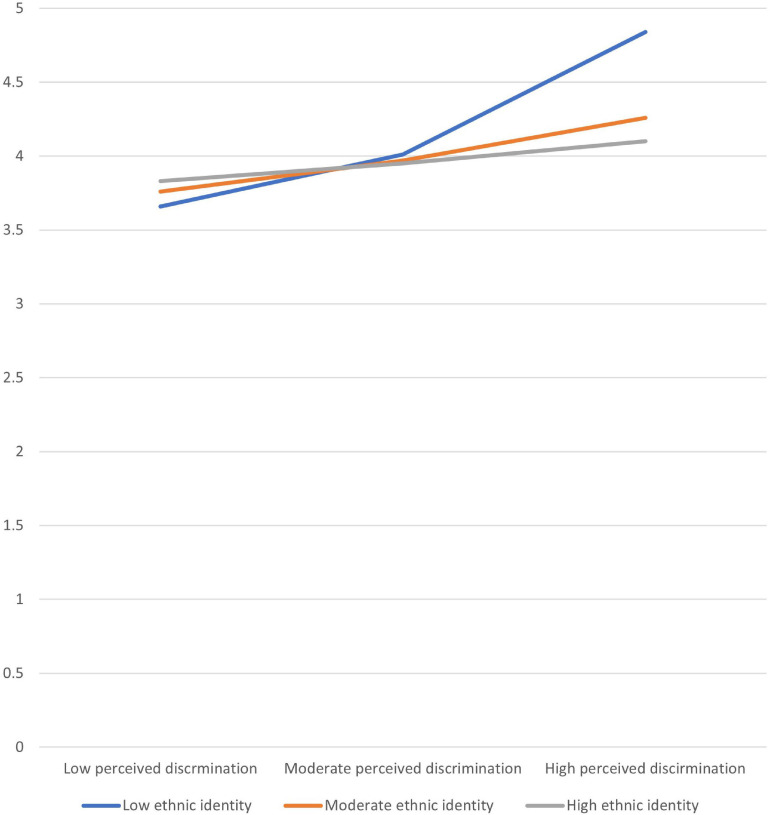
The interaction effect of perceived discrimination and ethnic identity on personal/career motivation.

To test the moderating role of ethnic identity in the relationship between perceived discrimination and personal/career-driven motivation, we used conditional process modeling as outlined by Hayes (2013) using the PROCESS macro. The procedure used bootstrapping with an SPSS application (PROCESS) developed by Preacher and Hayes (2004). PROCESS provides a method for probing the significance of conditional indirect effects at different values of the moderator. Age, high-school GPA, and SES served as the control variables. Testing the moderating role of ethnic identity in the relationship between perceived discrimination and personal/career-driven motivation showed that the model was significant, *F*(6,72) = 3.14, *p* = 0.009, accounting for 20.7% of the variance in personal/career-driven motivation. Age, high-school GPA, and SES did not have main effects on personal/career-driven motivation. Perceived discrimination and ethnic identity had significant effects on personal/career-driven motivation (*B* = 0.0.994, *SE* = 0.388, *p* = 0.012, and *B* = 0.0.532, *SE* = 0.215, *p* = 0.016, respectively). The product term of the interaction between perceived discrimination and ethnic identity was significant (*B* = −0.196, *SE* = 0.092, *p* = 0.036). That is, ethnic identity moderated the link between perceived discrimination and personal/career-driven motivation. *Post hoc* probing of the interaction revealed that the interaction was significant for low and moderate levels of ethnic identity (low ethnic identity, *B* = 0.342, *SE* = 0.106, *p* = 0.002; moderate ethnic Identity, *B* = 0.209, *SE* = 0.075, *p* = 0.007), but not for high ethnic identity (*B* = 0.11, *SE* = 0.081, *p* = 0.177). Figure 2 displays the interaction effect of perceived discrimination and ethnic identity on personal/career-driven motivation. As seen in Figure 2, the positive relationship between perceived discrimination and personal/career-driven motivation became stronger under lower levels of ethnic identity. However, in contrast to the direction of our third hypothesis, ethnic identity did not protect students against the adverse effects of discrimination. Rather, the strength of the positive association between discrimination and personal/career-driven motivation decreased with the levels of ethnic identity and became non-significant under high ethnic identity.

To test the moderating role of ethnic identity in the relationship between perceived discrimination and expectation -driven motivation, we used conditional process modeling (Hayes, 2013) with age, high-school GPA, and SES serving as the control variables.

Result showed that the model was significant, *F*(6,72) = 2.369, *p* = 0.038, accounting for 16.5% of the variance in expectation-driven motivation (Table 3). Age, high-school GPA, and perceived discrimination did not have main effects on expectation-driven motivation. However, SES had a main effect on this type of motivation (*B* = 0.283, *SE* = 0.123, *p* = 0.025) and so did ethnic identity (*B* = 0.615, *SE* = 0.283, *p* = 0.033). In contrast to the third hypothesis, the product term of the interaction between perceived discrimination and ethnic identity was not significant (*B* = −0.176, *SE* = 0.121, *p* = 0.152). Results are presented in Table 3.

**TABLE 3:** Results of the moderation analysis (by PROCESS Model 1).

Outcome variable	Independent variables	*B*	*SE*	*T*	*P*
Personal/career-driven motivation	Age	−0.004	0.020	−0.214	0.831
	GPA	0.002	0.044	0.442	0.660
	SES	−0.021	0.094	−0.231	0.818
	Perceived discrimination	0.994	0.388	2.564*	0.012
	Ethnic identity	0.532	0.215	2.477*	0.016
	Perceived discrimination × ethnic identity	−0.196	0.092	−2.136*	0.036

Expectation-driven motivation	Age	−0.033	0.026	−1.272	0.208
	GPA	−0.001	0.006	−0.211	0.834
	SES	0.283	0.123	2.294*	0.025
	Perceived discrimination	0.691	0.511	1.353	0.180
	Ethnic identity	0.615	0.283	2.174*	0.033
	Perceived discrimination × ethnic identity	−0.176	0.121	−1.449	0.152

*p < 0.05. **p < 0.01. ***p < 0.001. SES, socio economic status; GPA, grade point average.

## Discussion

The current study examined the relationship between perceived discrimination and motivation to pursue higher education among undergraduate students of Ethiopian origin. We also examined the role of ethnic identity as a potential factor that may attenuate this relationship. Results indicated that frequent experiences of discrimination were positively associated with personal/career-driven motivation to pursue higher education but not with expectation-driven motivation. Moreover, we found that ethnic identity moderated the positive effects of discrimination on personal/career-driven motivation so that the strength of this association decreased with the levels of ethnic identity.

The simple correlation analysis showed that ethnic identity may be linked to pursuing higher education through two separated motivational paths; a personal path and a family-social path. Ethnic identity may be related to personal/career-driven motivation because focusing on group affiliation enhances student’s overall self-esteem, self-confidence, and general aspirations (Forrest-Bank and Cuellar, 2018; van Bergen et al., 2019). Ethnic-identity achievement (e.g., high levels of exploration and commitment) may also reflect a more adaptive identity style, which is generally associated with better well-being and increased self-esteem in minority youth (Turnage, 2004; Umaña-Taylor et al., 2014). Second, ethnic identity and increased connectedness to ones’ family and community may be related to increased expectation-driven motivation because they motivate students to conform to group expectations and social pressures as a means of maintaining a positive social identity (Tajfel and Turner, 1979). Hence, students with high ethnic identity may be driven to pursue higher education and be successful in their studies due to family obligations, responsibility, and a sense of guilt related to the family (Tseng, 2004; Choi et al., 2014).

Our finding that perceived discrimination was positively associated with personal/career-driven academic motivation is surprising considering previous research about the adverse effects of discrimination (Alfaro et al., 2009; Walsh et al., 2018). However, there is evidence that discrimination may be also associated with increased motivation (Isik et al., 2018). For example, in a study of Latino students in the United States, Perreira et al. (2010) compared the academic motivation of Latino youth in an emerging Latino community with that of Latino youth in a traditional settlement community. Their results showed that youth in the emerging Latino community were more academically motivated despite greater fears of discrimination. The authors attributed these findings to several resilience factors, including being an immigrant, having a stronger sense of ethnic identification, and having a stronger sense of family obligation. These factors helped Latino youth develop a more positive view of school environments and counter the harmful effects of discrimination. It is therefore possible that increased awareness of discrimination and its harmful social, cultural, and financial effects lead minority students to combat it by investing extra efforts in schoolwork and proving their potential (Perreira et al., 2010).

The immigration background of Ethiopian students may also explain why discriminatory experiences are translated into increased efforts to enroll and succeed in academia. Studies of first-generation immigrant children have shown higher academic aspirations and expectations than their third-generation peers (Bohon et al., 2006). This “immigrant paradox,” or “second-generation advantage” (Kasinitz et al., 2008), was attributed to immigrant’s cultural capital and to parental beliefs, expectations, and values that favor higher education or focusing on hard work rather than social skills (Glick et al., 2009; Feliciano and Lanuza, 2017). Regarding Ethiopian immigrants, previous qualitative research (Ben Simon et al., 2018) pointed to the importance of parental values and expectations in students’ academic success. Parental influence included educational aspects (e.g., identifying education as important means for upward social mobility, exploring opportunities for extra curricula education, being a role model) as well as an emotional aspect (optimism, encouragement, and involvement). As participants for this study were recruited through self-selection, it is possible that they had enough personal and social support to resist the negative effects of discrimination throughout their life and therefore could use such experiences as a motivational resource. This self-selection sampling may also explain why, in contrast to previous research which associated perceived discrimination with poorer family relationship (Riina and McHale, 2010; Bécares et al., 2015) and lower parental expectations (Varner and Mandara, 2013), our results showed no significant associations between perceived discrimination and expectation-driven motivation.

Together, our results suggest that while perceived discrimination may motivate minority students to counter the harmful effects of discrimination by investing extra intellectual efforts and pursuing higher education, its association with family expectations and obligations would be more complex. As observed in the Ethiopian community, discrimination and microaggression are often directed toward communities with limited resources that have already experienced a severe disruption to their social fabric due to displacement, loss, trauma, and distrust (Silove et al., 2017). Therefore, individuals who experienced higher levels of discrimination would be more isolated and marginalized and less motivated to pursue higher education to conform with others’ expectations. Nevertheless, further research should be conducted with Ethiopian individuals who did not pursue higher education.

Testing the moderating role of ethnic identity in the relationship between perceived discrimination and the motivation to pursue higher education confirmed that Ethiopian ethnic identity moderated the effects of perceived discrimination on personal/career-driven motivation to pursue higher education but not on expectation-driven motivation. Unlike Phinney’s moderation hypothesis (2003), we found that the positive relationship (and not the negative relationship) between perceived discrimination and personal/career-driven motivation was significant under low and medium levels of ethnic identity, but not under higher levels. These findings suggest that ethnic identity may buffer the effects of perceived discrimination so that the facilitating effect of discriminatory experiences on personal/career-driven motivation diminishes under high levels of ethnic identity.

A possible explanation for the interaction effect between ethnic identity and perceived discrimination on personal/career-driven motivation is that awareness of discrimination may motivate students to pursue and excel in academic education only if they hold a more dynamic and flexible perspective of their ethnic identity. Such flexibility of the self may be particularly important for minority youth who must negotiate between different cultural contexts (Yeh and Hwang, 2000). However, if discrimination is coupled with intensive levels of ethnic centrality or with rigidity, students would strongly identify with their stereotyped and marginalized group (Pahl and Way, 2006; Brittian et al., 2015), thus “losing” the potentially motivating force of discrimination. In this regard, examining students’ identification with Israeli values (Israeli identity) may shed light on the complex relationship between discrimination, ethnic identity, and motivation. For example, it would be important to explore whether strong identification with Ethiopian culture is associated with low identification with Israeli culture and therefore, with increased marginalization.

Another possible explanation for the interaction effect is that the moderating role of ethnic identity may vary by specific ethnic identity dimensions (Quintana, 2007; Bombay et al., 2010). For example, discrimination was associated with negative mental-health outcomes for individuals with greater engagement with one’s ethnic group (behavioral dimension of ethnic identity), but not for individuals with clarity regarding the meaning and importance of one’s ethnic group (cognitive dimension of ethnic identity) (Lee et al., 2015). For example, Torres et al. (2011) demonstrated in samples of Latino adults that exploration is associated with greater psychological distress in response to discrimination whereas commitment served as a buffer. The lack of consensus about how discrimination and ethnic identity work together to impact psychological health, may be partly attributed to the diversity of ethnic or racial groups, ages, immigration background, measures, and outcomes. Therefore, further investigation of the effects of ethnicity, gender, SES, and developmental processes is required (Yip et al., 2008).

An alternative interpretation of our findings is related to a ceiling effect on personal/career-driven motivation. A ceiling effect might explain why students who reported high levels of ethnic identity, being already high on discrimination, did not experience the same increase in their academic motivation in response to higher discrimination as students with lower levels of ethnic identity. It is also possible that students who are consistently aware of their ethnic identity, and therefore have high motivation, may not be as sensitive to changes in their environment. However, among students whose ethnic identity is not always salient, it is expected that discrimination would be beneficial for motivation, as these students will respond to experiences of having their ethnic identity relevant (Douglass et al., 2016).

Several limitations should be noted when considering these findings. First, our results could be self- report biased, a bias more pronounced in ethnic minority groups (Rosenman et al., 2011). The present study is also limited in terms of the generalizability of the sample included. Because the sample consisted of students who voluntarily signed up to participate in psychological research, it is possible that participation was confounded with academic performance. Therefore, participating students may not be representative of the general population of students of Ethiopian origin. This study was also cross-sectional in nature and therefore cannot provide any information about causality. Longitudinal designs should be employed in future research to understand the effects of positive and negative academic climate (e.g., discriminatory or supporting) on different types of academic motivations, academic performance, and academic adjustment from enrollment in studies to graduation. Studying these variables longitudinally will provide a better understanding of the effect of ethnic-racial socialization within the context of racial discrimination. Furthermore, by employing longitudinal designs, we can identify when perceptions of racial discrimination are the most harmful and influential along the higher-education continuum (Banerjee et al., 2018). Finally, focusing on discrimination as a risk factor for academic motivation, the current study addressed the role of Ethiopian identity as a possible protecting factor. However, previous research in Israeli-Ethiopian youth showed that Israeli identity plays an important role in immigrant’s wellbeing (Walsh et al., 2012) and that examining both Ethiopian and Israeli identities may be of great value.

From a practical point of view, our findings suggest that both perceived discrimination and academic motivation are needed to be addressed in any effort to enhance academic adjustment and performance of ethnic minority students. Efforts should be made to facilitate an inclusive academic environment through educational programs, curricular and co-curricular activities, and employment of a diverse workforce (Hurtado et al., 2015). Bhopal and Pitkin (2018) advocated for mandatory unconscious bias training for academic personnel who are involved in admissions processes. Likewise, the University of California established a website where students can anonymously report incidents of discrimination and bias. An important aspect of enhancing academic diversity entails the uncovering of racist and gendered discourse in institutes of higher education. Reflecting upon her own experiences as a black woman professor, Henry (2015), suggested that in contrast to the academic ideals of social justice, many of her colleagues were completely unaware of the ways in which their attitudes and behaviors were perceived as racist by their non-white colleagues nor how the system of white supremacy is at work. Finally, helping ethnic minority students to develop motivation for learning and a strong sense of academic engagement may promote their academic adjustment and performance.

## Data Availability Statement

The raw data supporting the conclusions of this article will be made available by the authors, without undue reservation.

## Ethics Statement

The studies involving human participants were reviewed and approved by Department of Education, Ben-Gurion University. The patients/participants provided their written informed consent to participate in this study.

## Author Contributions

OV and LH recruited participants and performed statistical analyses. OS and TI contributed equally to the development of study design, integration of findings, and writing the manuscript. All authors contributed to the article and approved the submitted version.

## Conflict of Interest

The authors declare that the research was conducted in the absence of any commercial or financial relationships that could be construed as a potential conflict of interest.

## Footnotes

1 The terms *racial socialization* and *ethnic socialization* are used broadly to refer to the intergenerational transmission of information regarding race and ethnicity (Hughes et al., 2006; Francis et al., 2021). Historically, research on racial socialization focused on African American parents’ efforts to help their children understand racial barriers given systems of racial stratification in the United States (e.g., Boykin and Toms, 1985; Spencer and Markstrom-Adams, 1990). Research on ethnic socialization originated in the experiences of immigrant Latino, Asian, African, and Caribbean groups in the United States, and focused mainly on children’s cultural retention, identity achievement, and in-group affiliation facing pressure to assimilate to the dominant cultural group (Knight et al., 1993). Currently, however, the two concepts overlap and cover the same conceptual territory (Hughes et al., 2006).
